# A Medical Meditation

**Published:** 1903-12

**Authors:** 


### A MEDICAL MEDITATION.

IN this utilitarian age of progress we have almost come to the conclusion that the old saying "money is the root of all evil" is about four fourths correct. Physicians, just as other men of business, must live by means of their own labor, and it is therefore necessary for them to combine business methods with the practice of the medical science. Few physicians are there who have money enough to practice their profession simply for the love of science or suffering humanity, but they are flesh and blood just as other individuals, and are expected to pay their bills on the first of the month even if the previous month's practice has been almost entirely for charity. They must charge for their labors if they expect to live. And yet on the other hand the physician who has no love for his profession nor any desire to aid in its advancement, and who only practices it for the dollars and cents it can give him, is totally unfit to be recognized as the "true physician." We have no patience with those men who say that they practice only for the living they can get out of it. We believe that there is one attribute which should be possessed by every physician, and that is honesty. It is the one attribute which distinguishes the true physician from the quack, and enables him to have the full confidence of the community in which he lives. By the term honesty, we do not mean it in the criminal sense of stealing, but in its bearing towards ourselves and the patients we treat. Not only does a patient consult the physician because he believes him to have the necessary medical qualifications to give him professional advice, but also because he believes him to be honest in all his dealings. Too often, we are sorry to say, physicians dispense their medical knowledge and advice from a purely mercenary standpoint.

Let us illustrate: A patient consults a physician because of some imaginary ailment he believes to exist. He has read in the newspapers some quack advertisement describing various symptoms (?) of some disease, and like the large majority of humanity he finds there described just the very trouble with which he is afflicted. Probably all he needs is a straightforward, honest statement from the physician after a careful examination, that there is nothing the matter. In many cases this is all the treatment needed. But how often are there physicians who will humor such an individual into thinking that there is some real trouble, and who will cause him to run up a big medical bill, only in time to find himself no better and irreparably stamped with the "consulting-doctor-habit."

Take, for instance, an individual who imagines he has chronic dyspepsia, or catarrh of the stomach, that disease so prevalent in the columns of our daily newspapers.

Such are indeed a savory meal for the quack and for those physicians whose especial delight is to write prescriptions and thus keep filled the medicine closet at home. Do not understand us to mean that there are no real patients suffering with such complaints needing the proper medical attention. Far from it, but what we do mean is that there are many individuals who need less doctors and more hygiene.

Take another illustration. Almost nine tenths of the human race imagine that they have catarrh of the nose, or throat, and consequently they become victims of the "spray habit" either at home or at the office of some specialist. We have heard of individuals in this city, and they seem to be increasing every year, who are daily in attendance at the specialist's office for no other purpose than to be "sprayed out" at so much per spray, and we are told that there are some who go habitually as often as two, or even three times daily for such applications! When such a habit has become as firmly fixed as this, there is but one conclusion to reach and that is such individuals are becoming cocain fiends. The law takes firm hold upon drug stores which dispense "dope" to negroes, but there should be some law to stop the cocain habit by means of sprays. Show us an individual who has been the victim of the "spray habit" for months and we will show you a nose whose mucous membrane will be diseased if the habit is not stopped. It is very money-making to slam the door and say "next" to the waiting throng of snifflers, but as honest physicians we should enlighten the public against such wholesale destruction. In matters medical the public must be educated by the physicians, and such education will alone keep from our homes wide-spread pestilence and disease. It will not do for physicians to allow a mercenary spirit to insinuate itself in the practice of medicine. Make reasonable and just charges for professional work done, but do not let the idea of an extra dollar keep you from telling the patient the truth and doing for him only that which is actually necessary. The physician who would operate upon a patient simply for the fee when he knows absolutely that no benefit will result from the same is guilty of perjury. The patient is at the mercy of his physician, and if there is confidence in his medical adviser he will follow implicitly whatever directions are given. Let the physician be an honest medical adviser and "do unto others as he would they should do unto him." R.

